# Complete Genome Sequence of Staphylococcus epidermidis PH1-28, Isolated from the Forehead of a Hyperseborrheic Donor

**DOI:** 10.1128/MRA.00165-21

**Published:** 2021-03-04

**Authors:** Pascal Hilaire, Leslie Landemaine, Sandy Contreras, Hélène Blanquart-Goudezeune, Patricia Siguier, François Cornet, Hélène Chiapello, Valentin Loux, Cécile Clavaud, Stanislas C. Morand

**Affiliations:** a L’Oréal R&I, Tours, France; b L’Oréal R&I, Aulnay-sous-Bois, France; c GenoScreen, Lille, France; d CBI, LMGM, Université de Toulouse, UPS, CNRS, Toulouse, France; e Université Paris-Saclay, INRAE, BioinfOmics, MIGALE Bioinformatics Facility, Jouy-en-Josas, France; University of Rochester School of Medicine and Dentistry

## Abstract

We report the complete genome sequence of Staphylococcus epidermidis commensal strain PH1-28, isolated from the forehead of a healthy donor. The assembled 2.6-Mbp genome consisted of one chromosome and five plasmids. These data will provide valuable information and important insights into the physiology and metabolism of this skin flora microorganism.

## ANNOUNCEMENT

Staphylococcus epidermidis is a Gram-positive commensal *Firmicutes* bacterium ubiquitously present on human skin and mucous membranes (1, 2). S. epidermidis strains exhibit ambivalent roles; as an opportunistic pathogen, S. epidermidis is a common cause of nosocomial infections due to its capability to form biofilms on medical implants (3). In contrast, some commensal strains protect against skin colonization by pathogens or modulate the immune system (4, 5). We present here the complete S. epidermidis strain PH1-28 genome sequence, isolated after swabbing the forehead of a healthy hyperseborrheic donor.

S. epidermidis PH1-28 was isolated during a research protocol (study number ARC/COPEG/1125) on a tryptic soy agar plate and cultured with tryptic soy broth (TSB) medium at 37°C. Initial S. epidermidis taxonomic identification was deciphered by a BLASTn search on the nonredundant database v2.2.29+ (6) after 16S PCR followed by Sanger sequencing. High-molecular-weight genomic DNA was extracted from an overnight culture grown in TSB at 37°C using the Gentra Puregene kit (Qiagen). Sequencing was first performed on a MinION device with an R9.4.1 flow cell (Oxford Nanopore Technologies; rapid sequencing SQK-RAD004 library with 400 ng of DNA and 50-s tagmentation; base calling with Guppy v4.2.2 in 450_bps fast configuration). Long reads (12.9 Gbp; 1,265,187 reads; *N*_50_, 17,655 bp) were controlled with MinIONQC v1.4.1 (7) and filtered and trimmed using Nanofilt v2.6.0 (-q 7 -l 1,000), and adaptors were removed with Porechop v0.2.4 (8, 9). Then, sequencing on a HiSeq 2500 system (Illumina; 2-kb Nextera XT library) generated short reads, which were assessed with FastQC v0.11.5 (10) and cleaned with cutadapt v1.18 (11) and Prinseq v0.20.4 (12) (with the parameters -trim_qual_right 30 -trim_qual_type min -trim_qual_rule lt -trim_qual_window 7 -ns_max_n 0 -noniupac -min_qual_mean 30 -trim_left 15 -min_len 60), resulting in 11,094,664 reads (936 Mbp). Both short and long cleaned reads were *de novo* assembled using the Canu v2.0 (13), SPAdes v3.10.1 (14), and MaSuRCA v3.3.0 (PE = pe 600 50) (15) tools. The consensus assembly was manually curated and carefully verified using Bowtie 2 v2.1.0 (16), minimap2 v2.17 (17), and Geneious Assembler (Biomatters) by calculating the Reads Mapped Back to Contigs (RMBC) index (99.6 to 99.9% rate for the three tools). After assembly polishing with Pilon v1.23 (18), the chromosomal and plasmid coverage and circularity were validated with GeniousPrime software v2020.2.5. Finally, the chromosome was rotated to start at *dnaA*. The whole-genome median coverage depth reaches 4,828-fold. The assembled ungapped genome is 2,598,183 bp long (GC content, 32.05%), comprising one chromosome (2,468,349 bp) and five plasmids, pSE1 (51,568 bp), pSE2 (45,209 bp), pSE3 (28,218 bp), pSE4 (2,491 bp), and pSE5 (2,348 bp). pSE5 is identical to Staphylococcus aureus plasmid CP047868. Structural and functional annotations, carried out with comparisons using the Prokka v1.14.6 (19), RAST v0.11 (20), and PGAP v4.12 (21) tools, identified 19 rRNAs (6 operons [16S-23S-5S] with an extra 5S copy) and 61 tRNAs. This process pointed out 2,332 protein-coding sequences, 248 of which (11%) encode hypothetical proteins.

GC skew analysis using BRIG v0.95 (22) showed a clear chromosomal GC+/– region segmentation. Comparison with other S. epidermidis complete genome sequences using dRep v2.5.4 (23) based on average nucleotide identity (ANI) showed that the closest strain is ATCC 14990 (ANI, 0.994). PHASTER analysis (24) revealed that the chromosome comprises a ca. 40-kb locus corresponding to the *Siphoviridae* class I StB20 prophage (25, 26). Searches for mobile elements using ISsaga v2.0 (27) and ISEScan v1.5.4 (28) showed the presence of 43 transposases, 21 of which belong to the identical IS*110* family. The PH1-28 strain accumulation-associated protein aap does not contain the classical C-terminal collagen triple helix domain, and both massive *ebh* and *fmtB* protein-coding genes enclose internal stop codons. According to TADB v2.0 (29), plasmids pSE1 and pSE2 contain two and one type-II toxin-antitoxin clusters, respectively. Finally, the Abricate v1.0.1 tool showed that the PH1-28 strain does not carry the *blaZ* beta-lactamase gene present in the closely related ATCC 14990 strain (30). Default parameters were used for all software unless otherwise specified.

### Data availability.

The complete genome sequence is available in GenBank under the accession numbers CP066371 to CP066376, BioProject PRJNA662445, and BioSample SAMN16085533. The version described in this paper is the first version. Next-generation sequencing (NGS) raw reads have been deposited at the Sequence Read Archive under the numbers SRR13274404 and SRR13274405.
